# Extractable quantum work from a two-mode Gaussian state in a noisy channel

**DOI:** 10.1038/s41598-021-03752-4

**Published:** 2021-12-20

**Authors:** Marina Cuzminschi, Alexei Zubarev, Aurelian Isar

**Affiliations:** 1 Department of Theoretical Physics, National Institute for Physics and Nuclear Engineering, 077125 Magurele-Bucharest, Romania; 2 Faculty of Physics, University of Bucharest, 077125 Magurele-Bucharest, Romania; 3 Plasma Physics and Nuclear Fusion Department, National Institute for Laser, Plasma and Radiation Physics, 077125 Magurele-Bucharest, Romania

**Keywords:** Quantum physics, Thermodynamics

## Abstract

We study a Szilard engine based on a Gaussian state of a system consisting of two bosonic modes placed in a noisy channel. As the initial state of the system is taken an entangled squeezed thermal state, and the quantum work is extracted by performing a measurement on one of the two modes. We use the Markovian Kossakowski-Lindblad master equation for describing the time evolution of the open system and the quantum work definition based on the second order Rényi entropy to simulate the engine. We also study the information-work efficiency of the Szilard engine as a function of the system parameters. The efficiency is defined as the ratio of the extractable work averaged over the measurement angle and the erasure work, which is proportional to the information stored in the system. We show that the extractable quantum work increases with the temperature of the reservoir and the squeezing between the modes, average numbers of thermal photons and frequencies of the modes. The work increases also with the strength of the measurement, attaining the maximal values in the case of a heterodyne detection. The extractable work is decreasing by increasing the squeezing parameter of the noisy channel and it oscillates with the phase of the squeezed thermal reservoir. The efficiency mostly has a similar behavior with the extractable quantum work evolution. However information-work efficiency decreases with temperature, while the quantity of the extractable work increases.

## Introduction

The key problem of thermodynamics at its foundation was to design devices for work production using internal energy resources. The first phenomenological description of thermodynamical engines was provided by Sadi Carnot in 1824^1^. On the basis of the first and second laws of thermodynamics it was deduced that any engine requires a heat flux and should be connected to a hot and a cold thermal reservoirs. According to Sadi Carnot, the maximal efficiency of heat to work conversion is determined by the temperatures of the hot and cold reservoirs and it is always lower than the efficiency of an ideal thermal machine^2^.

The end of the XIX century was characterised by the fast gain of knowledge about the microscopical structure of matter. In particular, it was elaborated the kinetic molecular theory and made connection between the statistical physics and thermodynamics. Using the statistical physics approach it was shown the presence of fluctuations in temperature, density and other physical parameters in systems composed of a small amount of molecules^3^. If a fluctuation can be characterised by larger values of thermodynamical parameters than surrounding matter, then it stores a finite amount of energy^4,5^. Scientific debate about the absolute character of the second law of thermodynamics and physics of fluctuations gave rise to a novel concept of hypothetical devices for energy fluctuation harvesting^6,7^.

In 1929 Leo Szilard proposed a model of a single molecular engine connected to a single heat reservoir^8^. The principal component of a Szilard engine is a cavity with a single molecule inside. At first stage one introduces a mobile impenetrable membrane in the middle of the cavity. After that, a measurement is performed to determine in which part of the cavity the molecule is localised. Using the measurement result, a load is attached to the membrane to extract a work via an isothermal expansion at a constant temperature^7,9^.

To put his engine functionality in agreement with the second law of thermodynamics, Szilard assumed that getting and using information about the molecule position implies energy costs^10^. He deduced that any information dependent machine requires energy for its functionality. This idea was developed by Landauer, who asserted that by erasing the information about the system one loses the ability to extract work from it. In addition, Landauer calculated the maximal value of extractable work per bit of information about the system structure. From the Landauer principle, it follows that any classical Szilard engine cannot produce work, because all extracted work will be used to write the measurement results and it will be dissipated as heat after erasing the memory^11^. The discussions about the possibility of work extraction using a quantum Szilard engine are crucial for the study of quantum thermodynamics^12^.

Until the last decades of the XX century no experimental implementations of Szilard engine were possible, due to the weak development of micro-technologies and low precision of measurement techniques^13,14^. However, with the fast development of nanotechnologies^15–17^, enhancement of ion traps technology and implementation of the first quantum computers^18^, the design of the Szilard engine became a practical challenge. Besides the classical schemes of Szilard engine, associated with Maxwell demon, there were proposed memoryless models, where the measurement is used only to localise the particle in its part of the box^19,20^.

Another way to maximise the extractable work is by using quantum correlations^21^. According to Refs.^22,23^ two correlated Szilard engines allow to extract more work than a single engine. Likewise, in Ref.^9^ it is proven that a two particle quantum Szilard engine allows to extract a larger quantity of work than a classical one in the case of bosons, however less work is extracted in the case of fermions. These facts made attractive the study of bosonic multi-particle Szilard engines for nanoscale energy harvesting^24^.

In this paper we describe a two-particle quantum Szilard engine, determine its efficiency, and estimate the maximal work quantity that can be extracted if the initial state is partially destroyed during its temporal evolution. We simulate the engine by two correlated bosonic modes placed in a noisy channel, characterised by temperature and squeezing parameter. The time evolution of the extractable work is presented as a function of the parameters characterising the bosonic modes and the squeezed thermal environment. In Sec. “Quantum work”, there are described the differences between the classical and quantum work. Some notable examples that outline the contrast between these two notions are presented. The Szilard engine cycle, both for classical and quantum case, is depicted in Sec. “Szilard engine”. The Szilard engine cycle for a bipartite system is presented in detail. In Sec. “Dynamics of two bosonic modes in a Gaussian noisy channel” we describe the dynamics of two bosonic modes in a Gaussian noisy channel, and in Sec. “Extracted work and information-work efficiency” we describe the work extraction protocol and derive the expression of the extractable work^21^. Moreover, we derive the expression of the efficiency of the Szilard engine by using the von Neumann entropy for two-mode Gaussian states. Then we describe and discuss the obtained results. The extension of the notion of extractable work for non-Gaussian states is presented in Sec. “Generalization for non-Gaussian states”. Finally, in Sec. “Conclusions” we present the main results and ideas of this manuscript.

## Quantum work

The notion of work resides in the field of classical mechanics and thermodynamics. It does not belong to in the category of observables like energy, position and momentum because it defines a process, not an instantaneous state of the system.

We will start by defining work in thermally isolated classical systems. The energy of a system is described by its Hamiltonian $H(z,\lambda )$, where *z* is the phase space point and $\lambda$ is the force parameter that can change the system energy in correspondence with the force protocol $\Lambda =\{\lambda (t)|0\le t\le \tau \}$. In this way work can be implemented by or taken from the system. Work can be defined as the difference between the energies of the final and the initial states of the system^25,26^:

1 $$\begin{aligned} w=H[Z(\tau ,z_0),\lambda (\tau )]-H(z_0,\lambda (0)), \end{aligned}$$

where $Z(t,z_0)$ is the phase space point. Its initial value is $Z(0,z_0)=z_0$ and its evolution is determined by Hamiltonian dynamics ${\dot{Z}}=\{H(Z,\lambda (t)),Z\}_P$, with $\{,\}_P$ being the Poisson brackets.

We can also define work as the integral of the supplied power to or from the system:

2 $$\begin{aligned} w=\int dt\frac{\partial H[Z(t,z_0),\lambda (t)]}{\partial \lambda (t)}{\dot{\lambda }}(t). \end{aligned}$$

We can easily define the analogous to Eq. (1) definition for quantum work based on the two energy measurement approach^25,27^, which requires two projective measurements of energy. The interaction of the system with the measurement device should be taken into account in the quantum scenario^28^. In the classical case the back-action of the measurement apparatus can be considered arbitrarily small. For the quantum case attempts to make back-action of the measurement device small can cause limitations regarding the measurement precision.

During the measurements two eigenstates of energy are obtained and the work is given by:

3 $$\begin{aligned} w=e_m(\tau )-e_n(0), \end{aligned}$$

where $e_m$ and $e_n$ are the energies of the final and the initial states.

This equation is in agreement with fluctuation relations of Jarzynski^29^ and Crooks^30^.

However, in the general case we cannot give an equivalent definition to Eq. (2) because continuous observation of the system will cause quantum Zeno effect^31^ and the dynamics of the systems will become invariable with time.

An analysis of the extracted work and of the efficiency in the classical and quantum versions of the magnetic Otto cycle has been performed in Ref.^32^. The authors have reached the conclusion that the amount of extracted work and efficiency in the classical case is larger than or equal to those in the quantum case. They explained this fact by the equilibrium configuration in the classical case in comparison with the quantum case, where only equilibrium states correspond to the thermal reservoirs.

Quantum Carnot cycle exhibits the same efficiency as the classical one, according to the study^33^, because the quantum Carnot cycle has a calculable probability of being reversible, while in the classical case a complete cycle would require an infinitely long time, due to the fact that the ideal gas is brought quite easily out of equilibrium.

## Szilard engine

The Szilard engine was initially described as a single molecule engine^8,34,35^. *N* ideal identical particles are placed in a box. The length of the box is *L* and for simplicity only one dimension is considered. This system is put in contact with a thermal reservoir of temperature *T*. In the box is introduced an adiabatic wall at the position $l<L$, and after that the number of particles in the left compartment of the box is checked. If the numbers of particles in each compartment are not identical, then the work will be performed when the wall moves to the equilibrium position. To complete the thermodynamic cycle and return the system to its initial state we need to remove the adiabatic wall^34^.

Now let us consider one single particle in a box. An adiabatic frictionless wall is placed in the middle of the box and the particle is left to expand isothermally. The amount of extracted work in this case is^21^:

4 $$\begin{aligned} W_0=k_BT\ln (2)[1-H(X)], \end{aligned}$$

with $H(X)=-\sum _x p_x\ln (p_x)$ being the Shannon entropy of the particle position *x* distribution. To complete the thermodynamic cycle, the initial state of the system has to be restored. For this purpose, erasure work $W_{eras}$ has to be generated.

In this article we consider two correlated parties A and B separately placed, each in its own container. In this case the quantum correlations represent an additional resource for work exaction. The Szilard engine cycle is implemented in the following way. Party B is measured, and due to backaction some information about party A is obtained. After that party A reaches a new equilibrium. The work obtained in this process is^21^:

5 $$\begin{aligned} W(A|B)=k_BT\ln (2)[1-H(A|B)], \end{aligned}$$

where *H*(*A*|*B*) is the conditional entropy of the party A given the measurement of the party B. The correlated Szilard engine can extract more work because mutual information is non-negative, $I(A:B)=H(A)-H(A|B)\ge 0$, from which it follows that $W(A|B)\ge W_0$. In this case the system again has to be restored to the initial state in order to complete the cycle.

## Dynamics of two bosonic modes in a Gaussian noisy channel

We study the evolution of two bosonic modes in Gaussian noisy channels in the framework of theory of open quantum systems, by means of the Markovian Kossakovski-Lindblad master equation in the interaction picture for the density operator $\rho$ in natural units^36–39^:

6 $$\begin{aligned} \frac{d \rho }{dt}=\sum _{k=1}^{2}\frac{\lambda }{2}\left\{ (N_{k}+1){\mathscr {L}}[{\hat{a}}_{k}]+N_{k}{\mathscr {L}}[{\hat{a}}_{k}^\dagger ]-M_{k}^*{\mathscr {D}}[{\hat{a}}_{k}]-M_{k}{\mathscr {D}}[{\hat{a}}_{k}^\dagger ]\right\} \rho , \end{aligned}$$

with ${\hat{a}}_{k}^\dagger$ and ${\hat{a}}_{k}, k=1,2,$ being the creation and annihilation operators of the two bosonic modes. $\lambda$ is the overall damping rate, while $N_{k}$ and $M_{k}, k=1,2,$ represent the effective photon numbers and the squeezing parameters of the squeezed (phase sensitive) baths, respectively. At thermal equilibrium, i.e. for $M_{k} = 0$, the $N_{k}$ is the average number of thermal photons in the reservoir. Lindblad superoperators are ${\mathscr {L}}[{\hat{O}}]\rho =2{\hat{O}}\rho {\hat{O}}^\dagger -\rho {\hat{O}}^\dagger {\hat{O}}-{\hat{O}}^\dagger {\hat{O}}\rho$ and ${\mathscr {D}}[{\hat{O}}]\rho =2{\hat{O}}\rho {\hat{O}}-{\hat{O}}{\hat{O}}\rho -\rho {\hat{O}}{\hat{O}}$. The positivity of the density matrix imposes the constraints $|M_{k}|^2\le N_{k}(N_{k}+1)$.

Any bipartite Gaussian state is fully defined by its first and second order moment with elements:

7 $$\begin{aligned} \overline{X_i}= & {} \langle \hat{X}_i\rangle ,\nonumber \\ \sigma _{ij}= & {} \frac{1}{2}\langle (\hat{X}_i\hat{X}_j+\hat{X}_j\hat{X}_i)\rangle -\langle \hat{X}_i\rangle \langle \hat{X}_j\rangle ,~i,j=1,\dots ,4. \end{aligned}$$

The brackets $\langle \dots \rangle$ denote the quantum average and $\hat{X}=\left( \hat{q_1},\hat{p_1},\hat{q_2},\hat{p}_2\right)$ is the vector of the canonical variables of the considered system.

The evolution imposed by the master equation preserves the Gaussian character of the states and the temporal evolution for its covariance matrix is the following^39–41^:

8 $$\begin{aligned} \sigma (t)=e^{-\lambda t} \sigma (0) +(1- e^{-\lambda t})\sigma (\infty ), \end{aligned}$$

where $\sigma (0)$ is the covariance matrix of the initial Gaussian state and $\sigma (\infty )$ is the asymptotic covariance matrix (diffusion matrix), which is determined only by the bath parameters^42^:

9 $$\begin{aligned} \sigma (\infty )=\bigoplus _{k=1,2}\sigma _{k}(\infty ), \end{aligned}$$

with (we put $\hbar =1$)

10 $$\begin{aligned} \sigma _{k}(\infty )=\left( \begin{array}{cccc} \left( \frac{1}{2}+N_k+M_{kR}\right) /\omega _k&{}M_{kI}\\ M_{kI}&{}\left( \frac{1}{2}+N_k-M_{kR}\right) \omega _k\\ \end{array} \right) , \end{aligned}$$

where $M_{kR}$ and $M_{kI}, k=1,2,$ denote the real and imaginary parts of $M_{k}$, respectively, with

11 $$\begin{aligned} N_{k}= & {} n_{th,k}(\cosh ^2 R+\sinh ^2 R)+\sinh ^2 R, \end{aligned}$$

12 $$\begin{aligned} M_{k}= & {} -(2n_{th,k}+1)\cosh R\sinh R\exp {i\phi }, \end{aligned}$$

$n_{th,k}=\frac{1}{2}\left( \coth \left( \frac{\omega _{k}}{2T}\right) -1\right) , k=1,2$, are the average numbers of thermal photons (we put here Boltzmann constant $k_B=1$) and $\omega _{k}$ are the frequencies of the two modes. For the two reservoirs we take the same temperature *T*, squeezing parameter *R* and squeezing phase $\phi$.

## Extracted work and information-work efficiency

We consider a bipartite system $\mathrm {AB}$ evolving in time in Gaussian noisy channels, as described in the previous Section, in a Gaussian state of the modes ${\hat{a}}\equiv {{\hat{a}}_{1}}$ and ${\hat{b}}\equiv {{\hat{a}}_{2}},$ characterised by a covariance matrix of the block form^43,44^:

13 $$\begin{aligned} \sigma (t)=\left( \begin{array}{cc} \sigma _{a}(t)&{}\quad \sigma _{ab}(t)\\ \sigma _{ab}^{\mathrm{T}}(t)&{}\quad \sigma _{b}(t)\\ \end{array} \right) , \end{aligned}$$

where $\sigma _{a}$ and $\sigma _{b}$ are the covariance matrices of the two individual modes, and $\sigma _{ab}$ contains the correlations between the modes.

As said previously, any Gaussian state is fully defined by its first and second order moments. In our case the first moments of the canonical variables of the system are set to zero, since they are irrelevant for our purposes. Namely, the quantum work is determined by the correlations in the system, and information about the correlations is contained solely in the covariance matrix. We consider Gaussian measurements, performed by party $\mathrm {B}$ on his mode, of the form ${\pi }_b(X)=\pi ^{-1}{D}_b(X){\rho }^{\pi _b}{D}_b^\dagger (X)$^21^, where ${D}_b(X)=\exp (X{{\hat{b}}}^\dagger -X^*{{\hat{b}}})$ represents the displacement operator and ${\rho }^{\pi _b}$ is the density operator describing a pure Gaussian state whose covariance matrix is $\gamma ^{\pi _b}=R(\theta )\mathrm{{diag}}({\mu }/{2},\mu ^{-1}/{2})R(\theta )^{\mathrm{T}}$, with $\mu$ taking values in the interval $[0,\infty )$ and the rotation matrix expressed by using the *y*-Pauli matrix $\sigma _y$ in the following way: $R(\theta )=\cos \theta \mathrm{\mathbbm {1}}-i\sin \theta \sigma _y$. $\mu =0$ corresponds to a homodyne measurement and $\mu =1$ corresponds to a heterodyne one.

The conditional state of the mode ${{\hat{a}}}$ does not depend on the measurement result ${\pi }_b(X)$, i.e $\sigma _{a|X}^{\pi _b}\equiv \sigma _{a}^{\pi _b}$. The conditional state covariance matrix expression is given by^21,45^:

14 $$\begin{aligned} \sigma _a^{\pi _b}=\sigma _{a}-\sigma _{ab}(\sigma _{b}+\gamma ^{\pi _b})^{-1}\sigma _{ab}^{\mathrm{T}}. \end{aligned}$$

After the measurement the reduced state of the first mode ${\hat{a}}$ is out of equilibrium and party $\mathrm {A}$ can extract work from the thermal bath by letting his state to diffuse in the phase space^21^. The system prepared in the post-measurement state is in contact with the thermal reservoir and, for simplicity, we take the reference state as the time-local state $\sigma _{a}(t)$, therefore the extracted work is due to the measurement backreaction^21^. As the state is independent of the outcome, its average entropy is $\int dX p_X S(\sigma _{a|X}^{\pi _b})=S(\sigma _a^{\pi _b})$. Then following Eq. (4), the extractable work can be defined by^21^:

15 $$\begin{aligned} W=k_B T\left[ S(\sigma _a)-S(\sigma _a^{\pi _b})\right] , \end{aligned}$$

where $k_{B}$ is Boltzmann constant and *T* the temperature of the thermal reservoir.

To quantify the entropy of the conditional state (14), we use the Rényi entropy of order 2, $S_2(\rho )=-\ln \mathrm {Tr}(\rho ^2)$^46^, which in case of Gaussian states becomes a fully legitimate entropy functional given by the expression

16 $$\begin{aligned} S_2(\sigma _{ab})=\frac{1}{2}\ln (\mathrm {det}\sigma _{ab}). \end{aligned}$$

Then the expression of the work (15) becomes^21,47^

17 $$\begin{aligned} W=\frac{k_B T}{2}\ln \left( \frac{\det \sigma _a}{\det \sigma _a^{\pi _b}}\right) . \end{aligned}$$

We emphasize that in our case work *W* has to be addressed as the output of a suitable work-extraction protocol. The existence of a nonzero *W* proves the presence of classical correlations between the two parties $\mathrm {A}$ and $\mathrm {B}$^21^.

In Ref.^34^ the information-work efficiency of a Szilard engine was defined as the ratio of the extracted work to erasure work:

18 $$\begin{aligned} \eta =\frac{W}{W_{eras}}. \end{aligned}$$

Here, $W_{eras}$ is proportional to the information stored in the system:

19 $$\begin{aligned} W_{eras}=k_BT\ln 2^{H(P)}, \end{aligned}$$

where $H(P)=-\sum _{i=1}^nP_i\log _2P_i$ is the Shannon entropy associated with the probability $P_i$ distribution. We employ von Neumann entropy as the counterpart of the Shannon entropy^48^. In quantum mechanics the probability distributions are replaced by the density operators $\rho ,$ and the von Neumann entropy is given by:

20 $$\begin{aligned} S(\rho )=-\mathrm {Tr}(\rho \log _2\rho ). \end{aligned}$$

In particular, for a *n*-dimensional Gaussian state $\rho _{G}$ the von Neumann entropy becomes:^49^

21 $$\begin{aligned} S(\rho _G)=-\mathrm {Tr}(\rho _G\log _2\rho _G)=\sum _{j=1}^ns_V(\nu _j), \end{aligned}$$

where $\nu _j,j=1,\dots ,n,$ are the symplectic eigenvalues of the covariance matrix and

22 $$\begin{aligned} s_V(x)=\left( x+\frac{1}{2}\right) \log _2\left( x+\frac{1}{2}\right) -\left( x-\frac{1}{2}\right) \log _2\left( x-\frac{1}{2}\right) . \end{aligned}$$

For a two-mode Gaussian state the symplectic eigenvalues can be expressed in terms of the symplectic invariants^49^:

23 $$\begin{aligned} \nu _{\mp }^2=\frac{\Delta {\mp }\sqrt{\Delta ^2-4\det {\sigma }}}{2}, \end{aligned}$$

where the seralian is given by $\Delta =\det \sigma _a+\det \sigma _b+2\det \sigma _{ab}$.

As the initial state we choose a squeezed thermal state given by:

24 $$\begin{aligned} \sigma _{STS}=\left( \begin{array}{cccc} a&{}\quad 0&{}\quad c&{}\quad 0\\ 0&{}\quad a&{}\quad 0&{}\quad -c\\ c&{}\quad 0&{}\quad b&{}\quad 0\\ 0&{}\quad -c&{}\quad 0&{}\quad b \end{array} \right) , \end{aligned}$$

with the matrix elements:

25 $$\begin{aligned} a= & {} n_1\cosh ^2r+n_2\sinh ^2r+\frac{1}{2}\cosh 2r,\end{aligned}$$

26 $$\begin{aligned} b= & {} n_1\sinh ^2r+n_2\cosh ^2r+\frac{1}{2}\cosh 2r ,\end{aligned}$$

27 $$\begin{aligned} c= & {} \frac{1}{2}(n_1+n_2+1)\sinh 2r, \end{aligned}$$

where $n_1$ and $n_2$ denote the average thermal photon numbers of the two modes and *r* represents the squeezing parameter. For $n_1=n_2=0$ the squeezed thermal state becomes a squeezed vacuum state.

Our purpose is to determine the evolution in time of the work extracted by two local agents, or demons, A and B, who share a bipartite Gaussian state of modes ${{\hat{a}}}$ and ${{\hat{b}}}$. They check how much work party A can extract from a heat bath when only local Gaussian measurements are performed. In order to run an information engine, party A does not need to perform a measurement on his system and extract work from the recorded outcomes: A can exploit the backreaction induced by the Gaussian measurement ${\pi }_b$ performed by B on the joint state and A extracts mechanical work by letting its conditional state $\sigma _a^{\pi _b}$ expand, that is let its state to thermalise. This expansion can be converted into mechanical work^21^.

We consider general two-mode states in the block form (13), with the initial covariance matrix given by Eq. (24), corresponding to squeezed thermal states. The extractable work $W(\theta )$ depends on the measurement angle, therefore we shall consider the average work value $W= \frac{1}{2\pi }\int _{0}^{2\pi } d\theta W(\theta ).$ In all the following simulations we set the Boltzmann constant $k_{B}=1$ and the dissipation constant is taken $\lambda = 0.1$.

The information-work efficiency (18) is calculated for the work value averaged over the measurement angle. The value of $W_{eras}$ is obtained using Eqs. (19), (21) – (23). The erasure work taken for the efficiency calculations was considered maximal in time over the Szilard engine working cycle, to ensure that the system is reset to its initial state.

**Figure 1:**
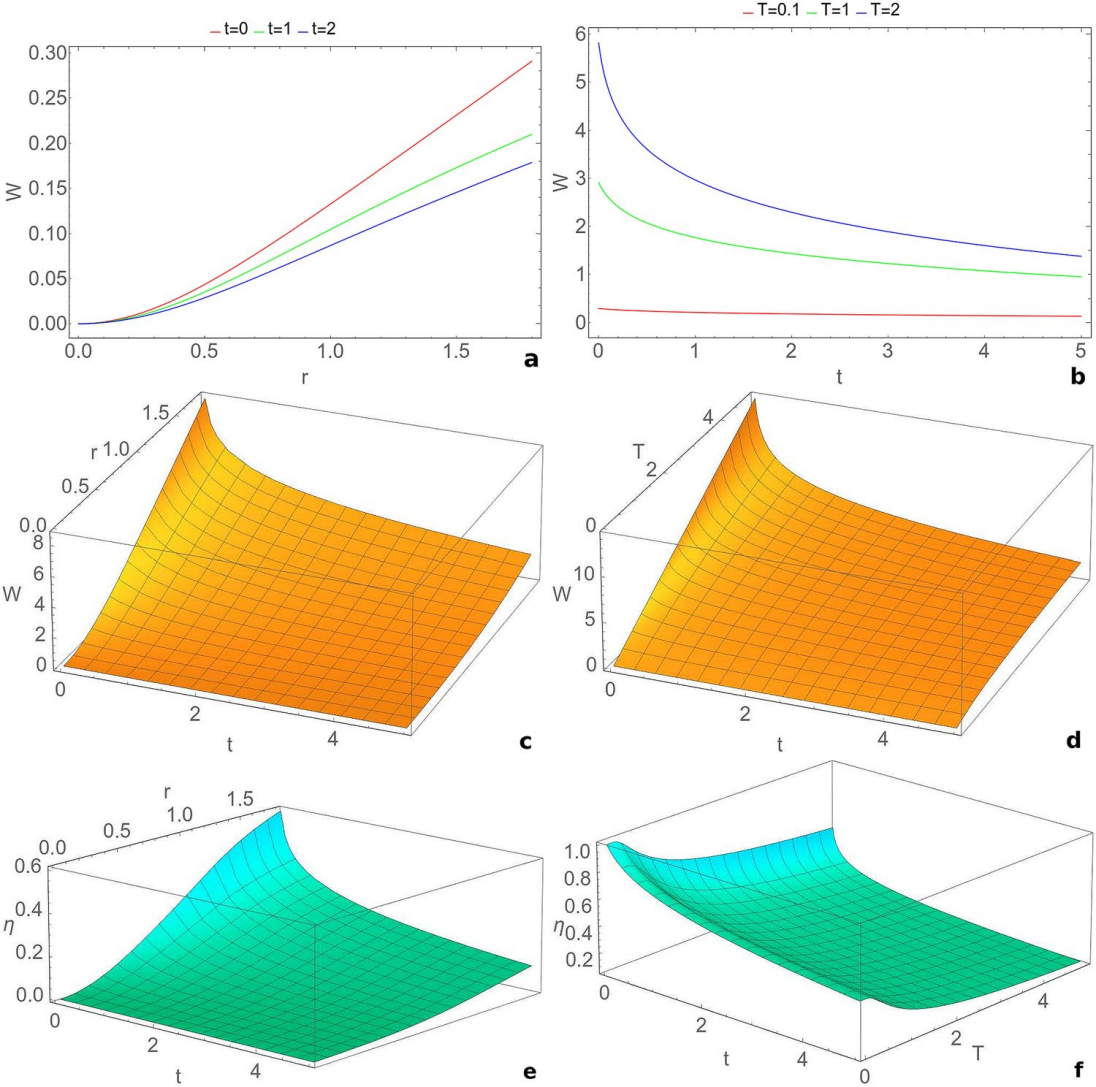
Evolution of the extractable work *W* averaged over the detection angle $\theta$ with: (**a**) squeezing *r* between modes at fixed moments of time $t=0$ (red), $t=1$ (green), $t=2$ (blue), for $T=0.1$, $\phi =\frac{\pi }{4}$, $\mu =0$; (**b**) time *t* for temperatures $T=0.1$ (red), $T=1$ (green), $T=2$ (blue), for $r=1.8$, $\phi =\frac{\pi }{4}$, $\mu =0$; (**c**) time *t* and squeezing *r* between the modes, for $T=3$, $\phi =0$, $\mu =1$; (**d**) time *t* and temperature of the reservoir *T*, for $r=1.8$, $\phi =\frac{\pi }{4}$, $\mu =0$. Evolution of efficiency of quantum work extraction with: (**e**) squeezing *r* between the modes and time *t*, for $T=3$, $\phi =0$, $\mu =1$; (**f**) time *t* and temperature *T* of the reservoir, for $r=1.8$, $\phi =\frac{\pi }{4}$, $\mu =0$. We consider the resonant case with $\omega _1=\omega _2=1$, an initial squeezed vacuum state ($n_{1} =n_{2} =0$) and $R=0.2$. This figure was obtained using Wolfram Mathematica 11.3.0^50^.

First we consider the resonant case, when both modes have the same frequency, and the initial state is a squeezed vacuum state. In Fig. 1 we illustrate the dependence of the extractable work and information-work efficiency by the Szilard engine cycle, on time, squeezing between the modes and bath temperature. The extractable work is averaged over the measurement angle. These parameters have a strong influence on the quantity of the extracted work. From Fig. 1a,c one can see that the extractable work increases with the squeezing between the modes and decreases in time. Large values of the squeezing between the modes correspond to an optimal functionality of the Szilard engine and better efficiency values (Fig. 1e). The engine has the best performance for strongly squeezed vacuum states and small times of evolution. In Fig. 1b,d it is represented the influence of bath temperature on the engine performance. For the whole range of the evolution times the extractable work averaged over the measurement angle increases with temperature. The best results are noticed for times very closed to the initial time. For long times the extractable work decreases due to the interaction with the thermal bath. However, the information-work efficiency is larger for small temperatures and for small times (Fig. 1f).

**Figure 2:**
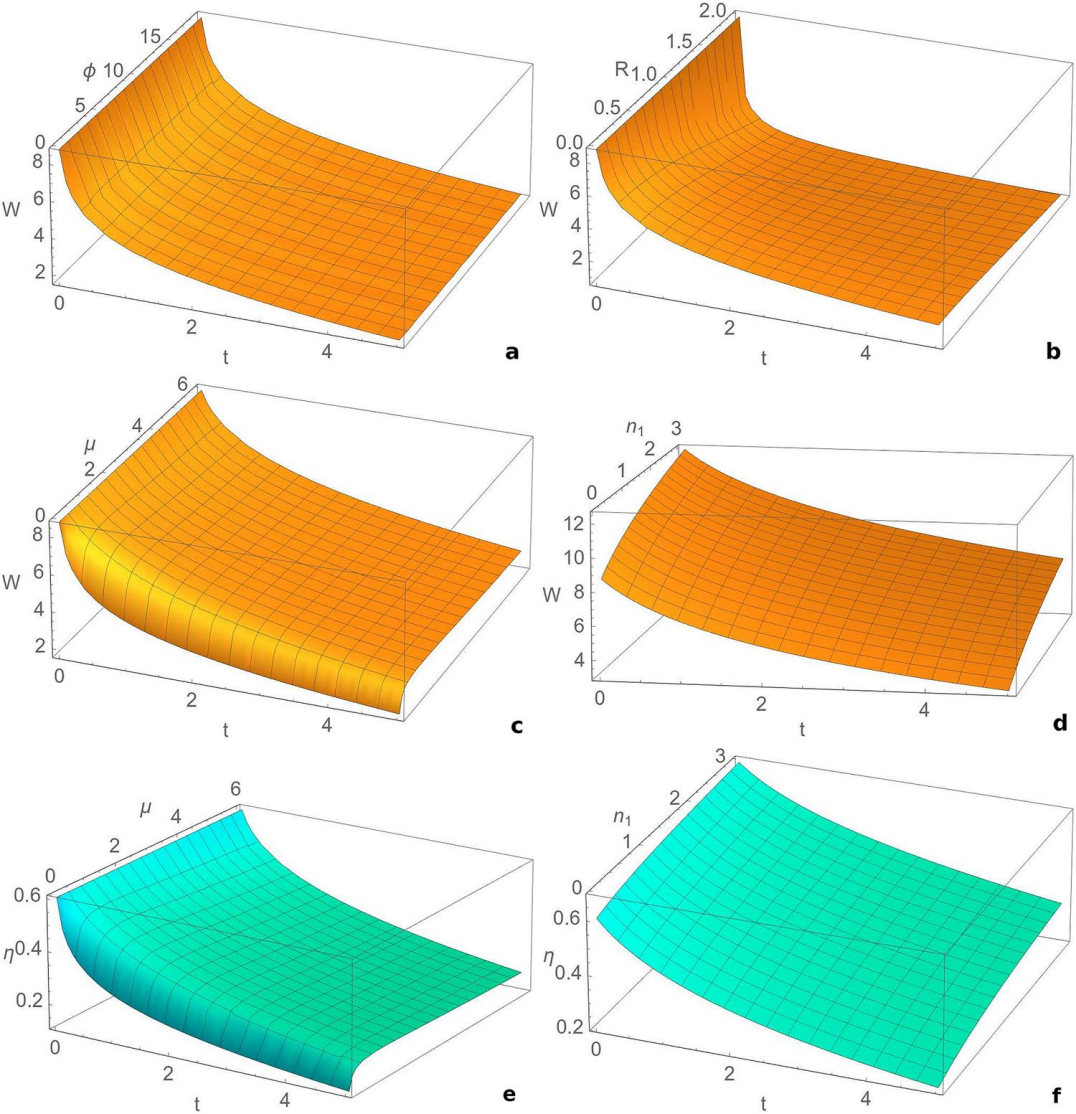
Evolution of the extractable work *W* averaged over the detection angle $\theta$ with: (**a**) time *t* and phase $\phi$ of the channel, for $n_{1} =n_{2} =0$, $R=0.2$, $\mu =0$; (**b**) time *t* and squeezing *R* of the bath, for $n_{1} =n_{2} =0$, $\phi =0$, $\mu =1$; (**c**) time *t* and strength of the measurement $\mu$, for $n_{1} =n_{2} =0$, $R=0.2$, $\phi =0$; (**d**) time *t* and the thermal photon number of the first mode $n_{1}$, for $R=0.2$, $\phi =\frac{\pi }{4}$, $n_{2} =0$, $\mu =0$. Evolution of efficiency of quantum work extraction with: (**e**) time *t* and strength of the measurement $\mu$, for $n_{1} =n_{2} =0$, $R=0.2$, $\phi =0$; (**f**) time *t* and the thermal photon number of the first mode $n_{1}$, for $R=0.2$, $\phi =\frac{\pi }{4}$, $n_{2} =0$, $\mu =0$. We consider the resonant case with $\omega _1=\omega _2=1$, $r=1.8$ and $T=3$. This figure was obtained using Wolfram Mathematica 11.3.0^50^.

From Fig. 2a we see that the extractable work is very weakly influenced by the phase of the bath and from Fig. 2b we notice that it slowly decreases by increasing the squeezing parameter of the bath. As already noticed, the extractable work decreases in time. We illustrate the influence of the strength of measurement on the extractable work in Fig. 2c, and on the information-work efficiency in Fig. 2e. We observe that the extractable work averaged over the rotational angle and the Szilard engine efficiency increase with the strength of the measurement and reach a maximum value for $\mu =1$, corresponding to a heterodyne detection, in agreement with the study^21^. Therefore, the heterodyne detection is optimal for work extraction, while the homodyne detection realizes a minimum device performance. The extractable work together with efficiency monotonically increase for $\mu \in [0,1]$ and monotonically decrease for $\mu \ge 1$. We studied also the influence of the thermal photon number on the extractable work. From Fig. 2d we notice that this quantity increases with the thermal photon number. Fig. 2f depicts the evolution of the Szilard engine efficiency with time and the thermal photon number of mode $\hat{a}$. We observe that the efficiency also increases with the thermal photon number, similar to the behaviour of the extractable work (Fig. 2d).

**Figure 3:**
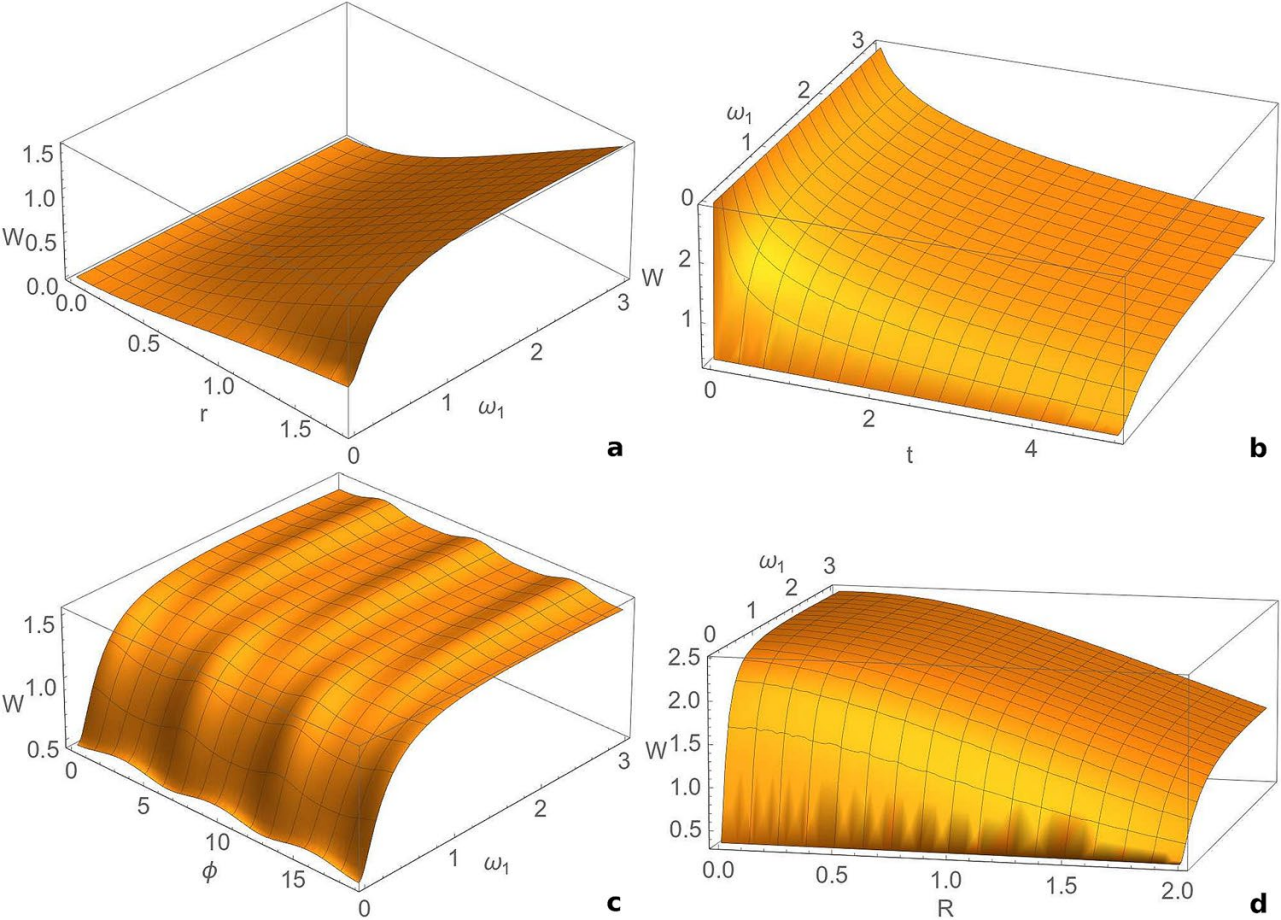
Dependence of the extractable work *W* averaged over the rotational angle $\theta$ on: (**a**) squeezing between modes *r* and frequency of the first mode $\omega _{1}$, for $t=2$, $R=0.2$, $\phi =\frac{\pi }{4}$; (**b**) time *t* and frequency of the first mode $\omega _1$, for $r=1.8$, $R=0.2$, $\phi =\frac{\pi }{4}$; (**c**) phase $\phi$ of the reservoir and frequency of the first mode $\omega _1$, for $t=2$, $r=1.8$, $R=0.2$; (**d**) squeezing parameter *R* of the bath and frequency of the first mode $\omega _1$, for $t=2$, $r=1.8$, $\phi =\frac{\pi }{4}$. We consider an initial squeezed vacuum state ($n_{1} =n_{2} =0$), $\omega _{2}=1, \mu =0, T=1$. This figure was obtained using Wolfram Mathematica 11.3.0^50^.

In the following we study the influence of the frequencies of the modes on the extractable work. In Fig. 3a we present the dependence of the extractable work averaged over the rotational angle on the frequency of the first mode and squeezing between the modes at some finite moment of time. We observe that for a weak squeezing the extractable work practically does not depend on the frequency. However, for a stronger squeezing the extractable work increases with the frequency and then it saturates. In Fig. 3b we show the dependence of the extractable work on time and the frequency of the first mode. Like previously, the extractable work quantity decreases in time and increases with the frequency. From Fig. 3c we observe that the extractable work has an weak oscillatory dependence on the phase of the squeezed thermal bath, with a period 2$\pi$. Finally, in Fig. 3d we illustrate the dependence of the extractable work on the environment squeezing and the frequency of the first mode, at some definite moment of time. Like previously, the quantity of extractable work decreases with the squeezing of the reservoir, but it increases with the frequency of the first mode, especially for relatively small values of this frequency, after which it reaches a plateau.

## Generalization for non-Gaussian states

In the case of Gaussian state and Gaussian measurments, the work extracted by party A is a direct measure of the one-way classical corelations ${\mathscr {I}}^{\leftarrow }$^21^. Let us consider a bipartite non-Gaussian state $\rho _{ab}$ with the von Neumann entropy $S(\rho _{ab})=-\mathrm {Tr}(\rho _{ab}\ln \rho _{ab})$. Party B again performs a measurement ${\pi }_b(X) \ge 0, \int dX {\pi }_b(X)=\mathbbm {1},$ which changes party A state due to backaction. The probability of getting the outcome *X* is $p(X)=\mathrm {Tr}(\rho _{ab}\mathbbm {1}_a\otimes {\pi }_b(X))$ and the state which belongs to party A collapses to a new state $\rho _{a|X}$. The classical correlations become ${\mathscr {I}}^{\leftarrow }=S(\rho _a)-\int dXp(X)S(\rho _{a|X})$. In this way, the general expression for work becomes^21^

28 $$\begin{aligned} W=k_BT[{\mathscr {I}}^\leftarrow (\rho _{ab})+S(\rho _a^{\mathrm {eq}})-S(\rho _a)], \end{aligned}$$

where $\rho _a^{\mathrm {eq}}$ is the equilibrium state of party $\mathrm {A}$ after thermalization with the reservoir. Eq. (28) can be rewritten as:

29 $$\begin{aligned} W=k_BT[{\mathscr {I}}^\leftarrow (\rho _{ab})+S(\rho _a||\rho _a^{\mathrm {eq}})+\Delta Q_a], \end{aligned}$$

where $S(\rho _a||\rho _a^{\mathrm {eq}})=\mathrm {Tr}(\rho _a\ln \rho _a-\rho _a\ln \rho _a^{\mathrm {eq}})$ and $\Delta Q_a$ is the heat absorbed in the isothermal expansion from the state before the measurement to the final state. When $\rho _a=\rho _a^{\mathrm {eq}}$ we reobtain the expression of the work (15), (17) for Gaussian states.

## Conclusions

By using as a Szilard engine a system composed of two entangled bosonic modes in a noisy channel, we investigated the evolution in time of the extractable work and information-work efficiency, as a function of the squeezing between the two modes, frequencies of the modes, their average numbers of thermal photons, temperature, squeezing and phase of the squeezed thermal reservoir, and strength of the measurement. The study has been performed based on the covariance matrix formalism. We have shown that the extracted work decreases in time. We have also shown that the quantity of extractable quantum work increases with the temperature of the reservoir and the squeezing between the modes, average numbers of thermal photons and frequencies of the modes. The work depends also on the strength of the measurement, attaining the maximal values in the case of a heterodyne detection. The information-work efficiency has a similar behaviour, excepting the evolution with the temperature. Namely, the efficiency decreases by increasing the temperature of the reservoir, while the quantity of extracted work increases with temperature. At the same time the extractable work is decreasing by increasing the squeezing parameter of the noisy channel and it oscillates with the phase of the squeezed thermal reservoir. The obtained results might find practical applications in the detection of quantum correlations, like quantum steering and entanglement, in bimodal bosonic systems.
